# CT-guided percutaneous approach for the treatment of peripheral pulmonary artery pseudoaneurysm: A case report

**DOI:** 10.1016/j.radcr.2021.07.031

**Published:** 2021-08-08

**Authors:** Rémi Grange, Flora Schein, Sylvain Grange

**Affiliations:** a Department of Radiology, Centre Hospitalier Universitaire de Saint-Etienne, Avenue Albert Raimond, Saint Priest En Jarez, Loire, France; b Department of infectious diseases, Centre Hospitalier Universitaire de Saint-Etienne, Avenue Albert Raimond, Saint Priest En Jarez, Loire, France

**Keywords:** Pulmonary arterial pseudoaneurysm, Glue, Embolization, CT

## Abstract

Pulmonary artery pseudoaneurysm is a rare but life threatening complication of pulmonary tuberculosis, considered as a diagnosis and therapeutic emergency. Transarterial embolization approach has become more widespread over the last few decades, and is now considered the first-line treatment over surgery. Percutaneous embolization under computed tomography (CT) or CT scan control has recently been reported by one centre as a first-line treatment for persistent peripheral Pulmonary artery pseudoaneurysm under certain conditions. We report the case of a 23-year-old female patient admitted in emergency for moderate haemoptysis, in a context of relapsing of tuberculosis. CT scan angiogram showed a peripheral pulmonary artery pseudoaneurysm of the lower left lobe, and persisted seven days later. After multidisciplinary meeting, a minimal invasive approach was decided. The patient was treated in first-line treatment by percutaneous transthoracic embolization, under CT-guidance, using N butyl-cyanoacrylate and Lipiodol mixture, without any complication. The percutaneous minimal invasive treatment seems to be a reliable approach to treat persistent peripheral pulmonary artery pseudoaneurysm.

## Introduction

Haemoptysis is a diagnostic and therapeutic emergency. Over 90% of haemoptysis is of bronchial origin [1]. The presence of a pulmonary arterial pseudoaneurysm (PAP) is a rare cause of haemoptysis, occurring in less of 10% of cases. The surgical approach was historical and gold standard treatment, but associated with high mortality and morbidity [2,3]. The minimally invasive endovascular approach has become the reference treatment, in first line, to treat PAP.

As this condition is rare, only case reports or small retrospective studies have been reported. However, for peripheral aneurysms, a percutaneous embolization using liquid agents (N- Butyl cyanoacrylate, thrombin, Onyx) has been reported as first-line treatment in few case reports [4,5] and a retrospective series of 27 patients [6], 24 of whom under CT guidance.

We report the case of a patient referred to our hospital for moderate haemoptysis in a context of relapsing tuberculosis. The CT scan angiogram showed a PAP of the lower left lobe, treated by percutaneous transthoracic approach, using N-butyl Cyanoacrylate (NBCA) and Lipiodol.

## Case presentation

A 28-year-old female patient came to the emergency room with dyspnea associated with moderate hemoptysis, with cough for 8 days and fever for 3 days. She has a past history of tuberculosis treated in 2013 and 2015, with poor compliance.

**Fig. 1:**
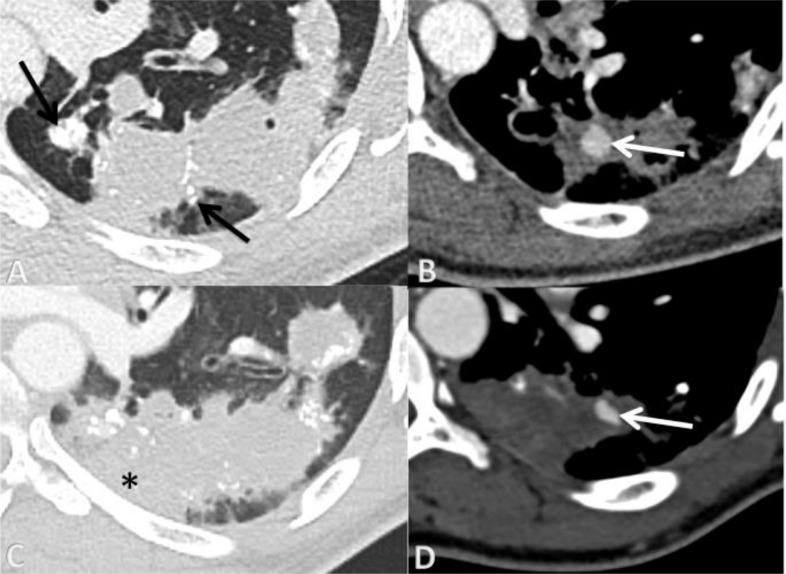
(A) Chest CT scan performed on the patient's emergency arrival in parenchymal section shows an alveolar condensation of the left lower lobe, with some nodular calcifications, suggestive of tuberculosis sequelae. (B) Axial chest CT angiogram shows a PAP of the left lower lobe measured at 9 × 9 mm, compatible with a PAP (whit arrow). (C) Chest CT scan in parenchymal section performed 7 days after arrival showed increased alveolar condensation of the left lower lobe (*). (D) Chest CT angiogram confirmed the persistence of a 4 × 9 mm pseudoaneurysm.

An emergency CT scan angiogram showed alveolar condensation of the left lower lobe, associated with a 9 × 9mm enhanced addition image, compatible with a PAP. The patient was monitored. However, the patient presented with a persistent moderate hemoptysis despite initiation of adapted regimen of tuberculosis. The CT scan performed 7 days later confirmed the persistence of a 4 × 9 mm PAP of the lower left lobe (Fig. 1). After multidisciplinary meeting, an embolization of this PAP was decided.

**Fig. 2:**
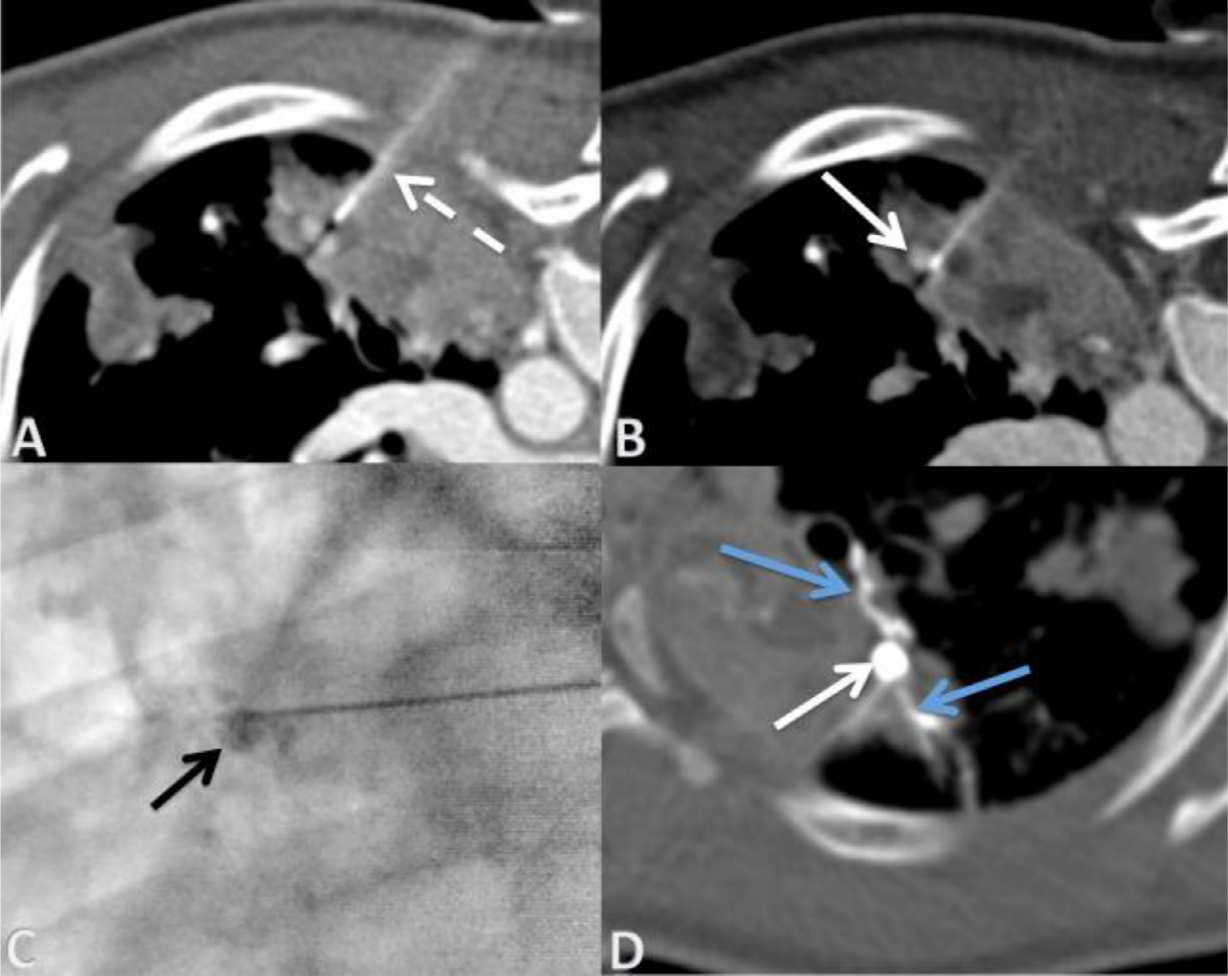
(A) After a CT scan angiogram, in procubitus, a 22G needle is inserted into the parenchyma, perpendicularly to the pleural surface, through the parenchymal condensation, in order to reduce the risk of pneumothorax (dotted arrow). (B) The per-procedure CT scan shows the correct positioning of the needle within the PAP (white arrow). (C) Scopic examination shows complete filling of the PAP (black arrow) after injection of a 0.5 cc mixture of Glubran2/Lipiodol 1/1. (D) The final CT scan demonstrates complete filling of the pseudoaneurysm (white arrow) and the artery feeding it (blue arrows). (Coloured version of the figure is available online).

Under general anaesthesia, the patient was first placed in procubitus position. A chest CT enhanced contrast acquisition confirmed the persistence of a 4 × 9mm PAP, without evidence of feeding artery (Fig. 2). A Chiba 22G-needle was placed step-by-step, within the PAP. A 1ml injection of contrast medium confirmed the correct positioning of the needle. After flushing the needle with 5% glocuse, the PAP was filled with a 0.5ml mixture of NBCA (Glubran2, GEM Srl, Viareggio, Italy) and Lipiodol (Guerbet, Villepinte, France) with a ratio 1/1. The CT scan performed without injection showed complete filling of the PAP and the feeding artery, without pneumothorax. CT scan angiogram confirmed the absence of filling of the PAP of alveolar haemorrhage Fig. 2. The total duration of the treatment was 30 minutes. She didn't have any significant recurrence of the hemoptysis, and will be discharged from the hospital 7 days later. She did not experiment any complication after procedure.

## Discussion

Massive haemoptysis is a diagnostic and therapeutic emergency, potentially life-threatening. 95% of massive haemoptysis is of bronchial origin, and less of 15% are of pulmonary arterial origin [7], secondary to PAP. Pulmonary artery pseudoaneurysm secondary to tuberculosis (Rasmussen aneurysm) is the main cause of PAP. Other main causes are represented by lung tumour, traumatism, iatrogeny, necrotic and fungal pneumonias [8]. The formation and development of a PAP results from the fragility of the arterial wall, by the traumatic, inflammatory, infectious or tumoral process, secondary to a destruction of the intima and the media.

Several treatment options are available. Emergency surgery, consisting of a wedge resection, lobectomy or pneumectomy, is the historic treatment, but associated with higher morbidity and mortality [2,3], with worse functional prognosis. Transarterial embolization (TAE) has gradually become prevalent, thanks to improved guidance, catheterisation and embolization equipment. It is now recognised as the first line treatment. Several embolization agents are available, including coils, plugs, liquid agents (NBCA, thrombin, Onyx) or covered stents. The choice depends on the size and location (proximal or distal) of the PAP, the size of the neck, the accessibility of the PAP, the need to keep the feeding artery patent, the experience of the habits of the operator.

However, for peripheral PAP, percutaneous embolization, using liquid agents (NBCA, thrombin, Onyx©) has been reported after normal angiography [4], technical failure [5,9] or incomplete treatment [11] in few case reports. Only one retrospective study of Lal et al [10] showed that the percutaneous approach is feasible as first-line treatment, without the necessity of pre-procedure angiography, in 27/39(69.2%) patients, with no major complication and a high clinical success rate (88.9%). This study lists 4 anatomical requirements for the percutaneous approach: a peripheral PAP, with a narrow neck, no fistulous communication either with bronchial and pulmonary system, no pneumothorax or emphysema on the biopsy pathway. This study reported only 1 minor complication, a glue migration in the bronchus. Shin *et al.*[11] proposed 4 classifications of PAP, after performing a diagnostic pulmonary and systemic arteriography. The percutaneous approach was performed for 2/24 patients with a type C (PAP only visualized at systemic angiography) or a type D (PAP not visualized at any systemic or pulmonary angiography) PAP, after failure of endovascular treatment, under ultrasound guidance, with clinical success. Compared to endovascular approach, percutaneous procedure is time saving, less expensive, limits patient's irradiation and injection of iodine contrast.

Percutaneous embolization using NBCA has been reported to be effective and safe to treat visceral [12] and soft-tissue pseudoaneurysms [13]. Liquid agents (thrombin, NBCA, Onyx) allow embolization of the aneurysm and the feeding artery in a single injection, with rapid thrombosis, including patients with coagulopathy. Another advantage is to modify the mixture's viscosity by changing the ratio of NBCA/Lipiodol, depending on the pseudoaneurysm's size and the risk of nontarget embolization. In our case and those reported by Lal *et al.*[6], a high viscosity was preferable (ratio 1/1), because of the small size of the pseudoaneurysm, and the risk of nontarget embolization. However, its injection technique can be tricky, subject to a progression curve.

A multidisciplinary approach is essential to adapt the treatment to the clinical state of the patient, and the characteristics of the PAP. The percutaneous minimal invasive treatment seems to be a reliable approach to treat persistent peripheral PAP. We believe that this approach should be performed as a first-line procedure, whenever possible, for persistent peripheral PAP. Retrospective studies with more selected patients are needed to evaluate the safety of such an approach.

## Patient consent

Informed consent for patient information to be published in this article was obtained.
